# Differentiation of Adrenal Adenomas from Non-Adenomatous Lesions: Diagnostic Value of Unenhanced Spectral CT

**DOI:** 10.3390/tomography12050068

**Published:** 2026-05-12

**Authors:** Tommasa Catania, Grazia Morabito, Simone Barbera, Massimo Venturini, Federico Fontana, Eduardo Maccarrone, Grazia Maria Arillotta, Velio Ascenti, Silvio Mazziotti, Thomas Joseph Vogl, Giovanni Foti, Tommaso D’Angelo, Giorgio Ascenti

**Affiliations:** 1 Diagnostic and Interventional Radiology Unit, BIOMORF Department, University Hospital “Policlinico G. Martino”, 98124 Messina, Italy; catania.tommasa@hotmail.com (T.C.); simobarbera7@gmail.com (S.B.); maccarrone.eduardo@gmail.com (E.M.); arillottagraziamaria@gmail.com (G.M.A.); silvio.mazziotti@unime.it (S.M.); tommaso.dangelo@unime.it (T.D.); giorgio.ascenti@unime.it (G.A.); 2 Diagnostic and Interventional Radiology Unit, Circolo Hospital, ASST dei Sette Laghi, University of Insubria, 21100 Varese, Italy; massimo.venturini@uninsubria.it (M.V.); federico.fontana@uninsubria.it (F.F.); 3 Department of Radiology, Policlinico Universitario, University of Milan, 20133 Milano, Italy; velio.ascenti@unimi.it; 4 Department of Diagnostic and Interventional Radiology, University Hospital Frankfurt, 60590 Frankfurt am Main, Germany; t.vogl@em.uni-frankfurt.de; 5 Department of Radiology, IRCCS Sacred Heart Hospital Don Calabria, 37024 Negrar, Italy; giovanni.foti@sacrocuore.it

**Keywords:** adrenal incidentaloma, dual-energy CT, spectral CT, virtual monoenergetic imaging

## Abstract

Background: Differentiating adrenal adenomas from non-adenomatous lesions remains a critical challenge in the management of adrenal incidentalomas. Conventional unenhanced CT relies on attenuation thresholds of 10 HU and 20 HU, which present trade-offs between sensitivity and specificity. Objectives: To evaluate the diagnostic performance of unenhanced Spectral CT using the attenuation difference between 40 keV and 140 keV virtual monoenergetic images for differentiating adrenal adenomas from non-adenomatous lesions. Methods: In this retrospective single-center study, 60 patients with adrenal lesions who underwent unenhanced dual-energy CT were included. Mean attenuation values were measured on conventional images and on virtual monoenergetic images at 40 keV and 140 keV. The spectral attenuation difference (Δ40–140 keV) was calculated. ROC analysis was performed to determine the optimal threshold and diagnostic performance. Additional analyses included DeLong comparison of correlated ROC curves and bootstrap resampling to estimate 95% confidence intervals for the area under the curve. Results: Forty-nine lesions were adenomas and eleven were non-adenomatous. The optimal threshold for Δ40–140 keV was −17 HU. When evaluated as a continuous variable, Δ40–140 keV yielded an area under the curve of 0.940 (95% confidence interval: 0.851–1.000), compared with 0.939 (95% confidence interval: 0.870–0.992) for conventional unenhanced attenuation. DeLong comparison showed no statistically significant difference between the two curves (*p* = 0.980). Diagnostic performance was as follows: HU ≤ 10 (AUC 0.816, diagnostic accuracy 0.70), HU ≤ 20 (AUC 0.883, diagnostic accuracy 0.87), and Δ40–140 keV ≤ −17 HU (AUC 0.940, diagnostic accuracy 0.90). The spectral attenuation difference demonstrated the highest overall diagnostic accuracy. Conclusions: Unenhanced Spectral CT using Δ40–140 keV improves discrimination between adrenal adenomas and non-adenomatous lesions compared with conventional attenuation thresholds. This technique may reduce indeterminate findings and limit the need for additional imaging.

## Simple Summary

Accurate differentiation between adrenal adenomas and non-adenomatous lesions is essential for appropriate patient management, yet it remains challenging with conventional imaging. Spectral computed tomography is increasingly used in clinical practice and provides additional quantitative information beyond standard Hounsfield Unit analysis. In this study, we evaluated whether specific spectral imaging parameters can improve lesion characterization. Our findings show that changes in attenuation values between low and high keV can enhance diagnostic accuracy compared with traditional thresholds. These results may help reduce unnecessary follow-up examinations or invasive procedures and support more confident decision-making, while also encouraging further research in larger prospective cohorts.

## 1. Introduction

According to the Society of Abdominal Radiology, an adrenal incidentaloma is defined as an incidentally detected adrenal nodule or mass unrelated to the clinical indication for the imaging examination performed [1]. More recently, a refined definition has been proposed, restricting adrenal incidentalomas to lesions measuring ≥1 cm and excluding patients with current or prior extra-adrenal malignancy [2].

Adrenal incidentalomas are relatively common, with a reported prevalence of approximately 3–7% in the adult population, and the majority represent benign, non-functioning adenomas. Accurate characterization of adrenal lesions as benign or malignant is therefore crucial, as imaging findings directly influence patient management and follow-up strategies [3,4,5].

Historically, CT characterization of adrenal lesions has relied on two main principles. First, an unenhanced attenuation value of ≤10 Hounsfield units (HU) has been considered diagnostic of lipid-rich adenomas, obviating the need for further imaging. Second, lesions with attenuation values >10 HU have traditionally been evaluated using contrast-enhanced washout techniques to differentiate lipid-poor adenomas from non-adenomatous lesions, with commonly accepted thresholds of ≥60% for absolute washout and ≥40% for relative washout [5,6,7].

More recently, an unenhanced attenuation threshold of >20 HU was proposed, in order to increase sensitivity in detecting benign lesions [2]. In parallel, dual-energy CT (DECT) has emerged as a promising technique for non-invasive tissue characterization beyond conventional HU measurements, exploiting the energy-dependent attenuation properties of different materials [8,9,10,11].

In particular, unenhanced dual-energy CT allows spectral attenuation analysis using virtual monoenergetic images (VMIs). The presence of intracellular lipids within adrenal adenomas results in a characteristic decrease in attenuation at lower energy levels, potentially providing incremental diagnostic information, even in lesions with attenuation values above 10 HU [12,13,14,15,16,17].

Based on previous evidence [18], we hypothesized that the attenuation difference between low- and high-energy VMIs (40–140 keV) could serve as a reliable discriminative parameter for differentiating adrenal adenomas from non-adenomatous lesions.

The aim of this study was to evaluate the diagnostic performance of unenhanced spectral CT for diagnosis of adrenal adenomas, in comparison with conventional unenhanced ≤10 HU and ≤20 HU cut-offs.

## 2. Materials and Methods

### 2.1. Study Population

Adult patients with adrenal nodular lesions who underwent unenhanced abdominal dual-energy Spectral CT between September 2021 and December 2025 were retrospectively identified from the institutional database of our university hospital. CT examinations were performed for various clinical indications, including both incidentally detected and clinically suspected adrenal lesions.

**Figure 1:**
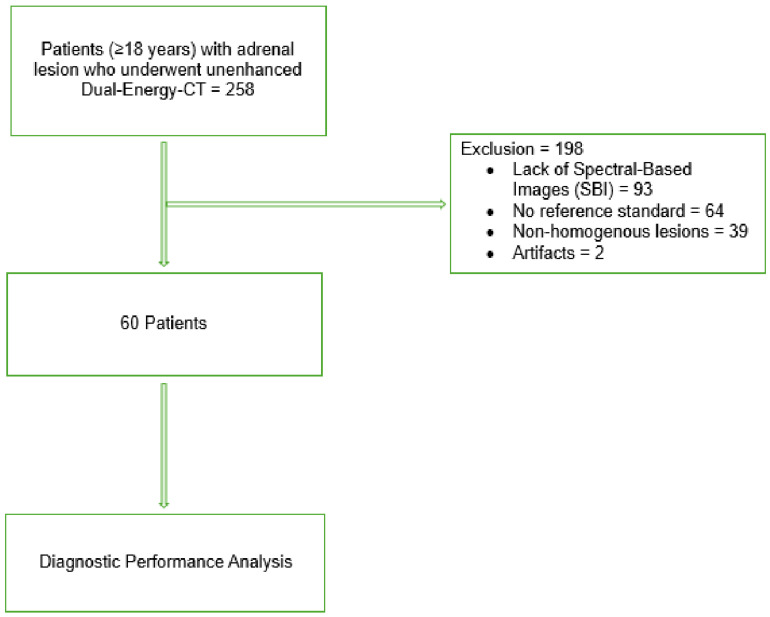
Flowchart of inclusion and exclusion criteria.

Inclusion and exclusion criteria are summarized in Figure 1.

The reference standard for all adrenal lesions was based on histopathologic examination, and/or interval imaging follow-up. Lesions showing no change in size for at least 12 months were considered benign adenomas, whereas lesions demonstrating a size increase greater than 20% in maximum diameter were considered non-adenomatous.

### 2.2. Spectral CT Image Acquisition

All images were acquired using dual-layer spectral-detector CT (IQon, Philips Healthcare, Eindhoven, The Netherlands), which provides both conventional (120 kVp) and spectral-based images.

Technical parameters are reported in Table 1.

Axial, unenhanced conventional 120 kVp and VMIs at 40 keV and 140 keV were reconstructed with contiguous 2 mm thick sections.

Conventional images were reconstructed using an iterative reconstruction algorithm (iDose 4, level 3; Philips Healthcare), and VMIs were reconstructed using a dedicated spectral image reconstruction algorithm (Spectral, level 3; Philips Healthcare).

### 2.3. Image Analysis

All image data were postprocessed using the proprietary workstation (IntelliSpace Portal, version 9.0; Philips Healthcare).

Image analysis was conducted by two board-certified radiologists in consensus, both blinded to clinical information and final diagnosis.

To measure mean unenhanced attenuation on conventional images and VMIs, a circular region of interest (ROI) was placed manually within the center of each adrenal lesion, avoiding surrounding fat or normal adrenal parenchyma.

For each lesion, measurements from three separate ROIs were averaged to ensure data consistency. Image analysis was based on consensus placement of the region of interest by two radiologists.

The spectral attenuation difference (Δ40–140 keV) was calculated as the difference between attenuation values measured on 40 keV and 140 keV VMIs using an identical ROI.

The spectral attenuation curve was also reconstructed from the VMIs at different energy levels.

### 2.4. Statistical Analysis

Statistical analyses were performed by using Med Calc software (MedCalc Statistical Software version 23.0.8 (MedCalc Software Ltd., Ostend, Belgium) and Matlab (Matlab, MathWorks v. R2024b, Natick, MA, USA)). Group comparisons were performed using the Mann–Whitney U test. Receiver operating characteristic analysis was conducted to assess diagnostic performance and determine optimal thresholds using the Youden index.

In addition, receiver operating characteristic curve analysis was performed for the continuous spectral attenuation difference (Δ40–140 keV) and for continuous conventional unenhanced attenuation values. Pairwise comparison of the corresponding areas under the curve was performed using the DeLong test for correlated receiver operating characteristic curves. Internal validation of the area under the curve estimates was performed by bootstrap resampling, with calculation of 95% confidence intervals.

## 3. Results

### 3.1. Patient Demographics

A total of 258 patients were initially identified. After application of exclusion criteria, 60 patients were included in the final analysis, thirty-one males and twenty-nine females, with a mean age of 66 years. Forty-nine lesions were adenomas and eleven were non-adenomatous lesions: metastases (8), pheochromocytomas (2) and adrenal carcinoma (1).

### 3.2. Diagnostic Performance Figures, Tables and Schemes

ROC curve analysis demonstrated that the optimal threshold to discriminate adenomas from non-adenomas, using Δ40–140 keV, was −17 HU, with values below this cutoff indicative of benign lesions.

When evaluated as a continuous variable, Δ40–140 keV showed an area under the curve of 0.940, with a bootstrap-derived 95% confidence interval of 0.851–1.000. Continuous conventional unenhanced attenuation showed an area under the curve of 0.939, with a bootstrap-derived 95% confidence interval of 0.870–0.992. Pairwise comparison of the two receiver operating characteristic curves using the DeLong test did not show a statistically significant difference (*p* = 0.980).

The diagnostic performance of all three parameters was high: HU ≤ 10 (AUC = 0.81), HU ≤ 20 (AUC = 0.88), and Δ ≤ –17 (AUC = 0.90). These values indicate good discriminative ability between benign and malignant lesions.

**Figure 2:**
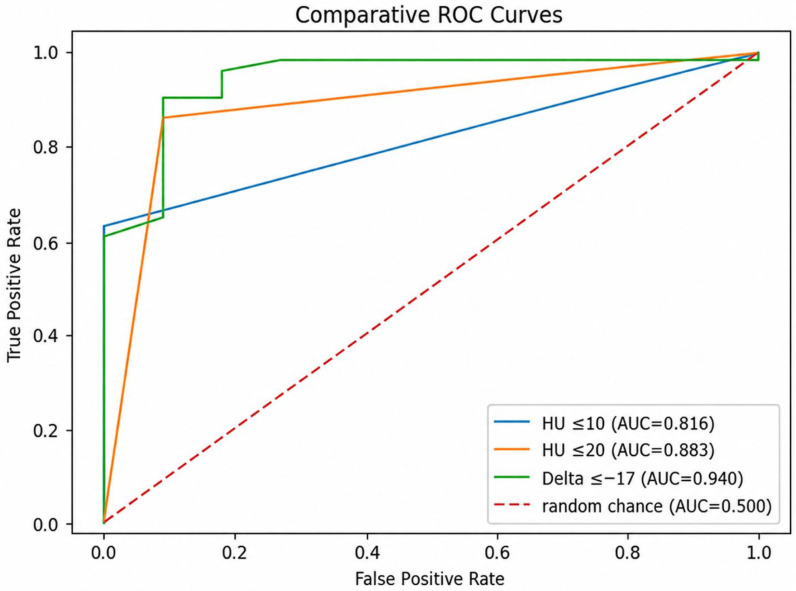
Result of receiver operating characteristic analysis.

The diagnostic performance of the spectral attenuation difference was superior to that of conventional attenuation thresholds, with the highest area under the curve (Figure 2).

**Figure 3:**
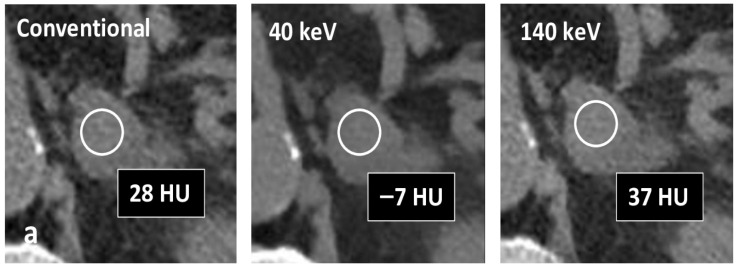

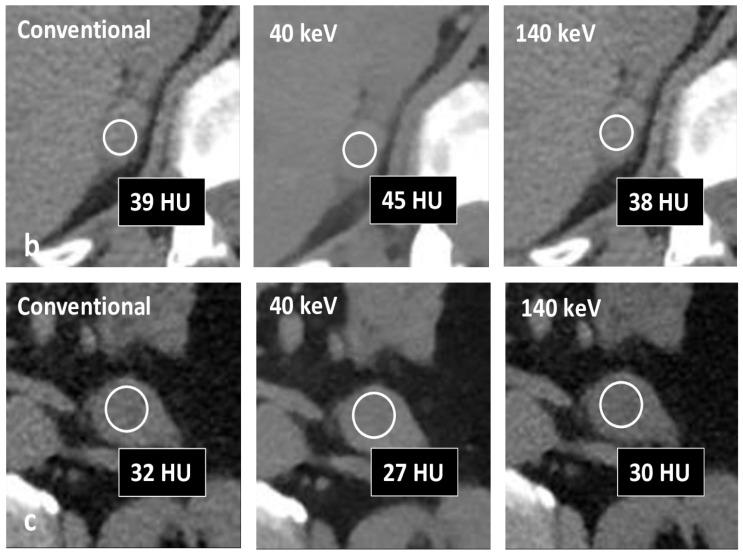
Axial unenhanced Spectral CT images of three adrenal masses indeterminate at conventional CT (HU > 20). (**a**) Lipid-poor adenoma; (**b**) metastasis from lung cancer; (**c**) pheochromocytoma. Lesion (**a**) shows decreased attenuation at 40 keV with Δ40–140 keV of −44 HU. Lesions (**b**) and (**c**) show no significant attenuation differences between 40 keV and 140 keV.

**Figure 4:**
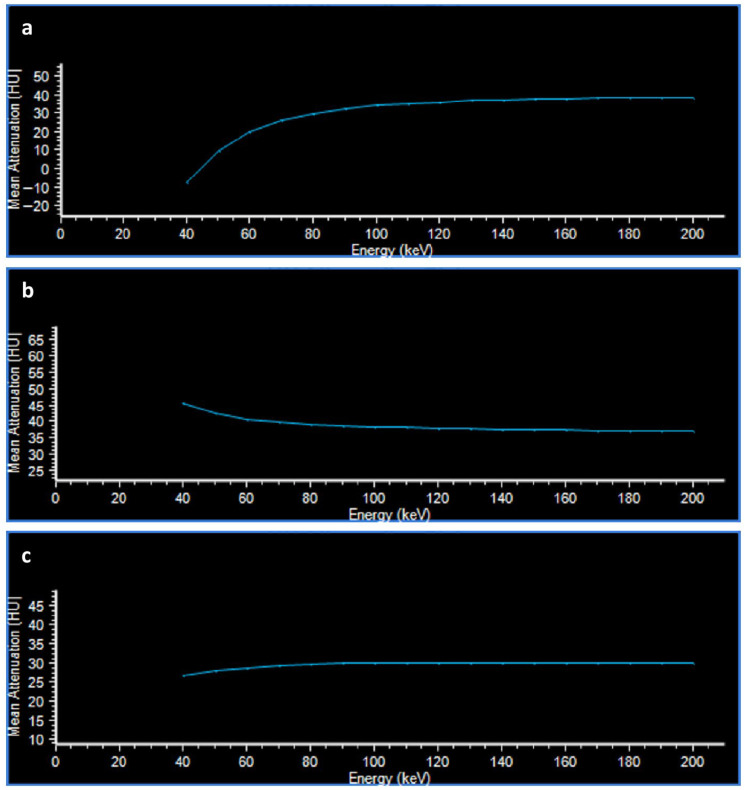
HU Attenuation Plot. The attenuation curves reflect the behavior of the same lesions in Figure 3 at different energy levels (keV). The curve of lesion (**a**) (lipid-poor adenoma) decreases at low energies, the curve of lesion (**b**) (adrenal metastasis) increases at low energies, while the curve of lesion (**c**) (pheochromocytoma) is approximately straight.

Significant differences were observed between benign and malignant lesions across all evaluated parameters (*p* < 0.0001). Adenomas exhibited lower unenhanced attenuation values and more negative spectral attenuation differences compared with non-adenomatous lesions (Figure 3 and Figure 4).

**Figure 5:**
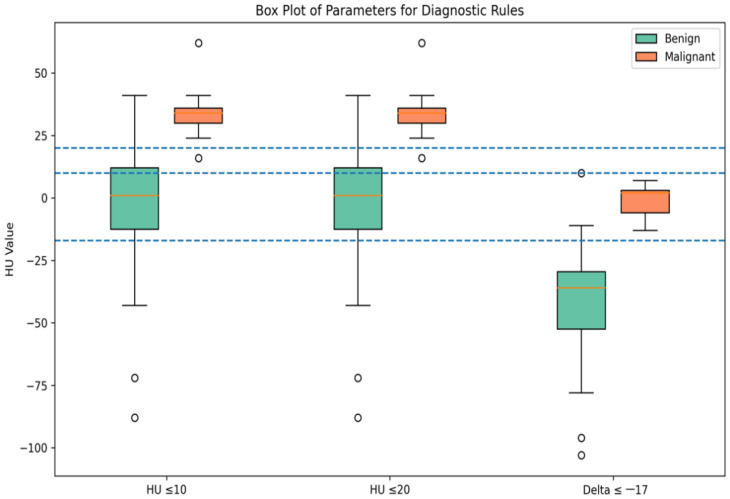
The box plot compares the distribution of three continuous radiological parameters between benign and malignant adrenal lesions. The blue dashed lines indicate the diagnostic cut-off values used in the analysis, corresponding to HU ≤ 20, HU ≤ 10 and Δ ≤ −17 HU thresholds for distinguishing benign from malignant lesions.

The results of the quantitative analysis are shown in Figure 5.

**Figure 6:**
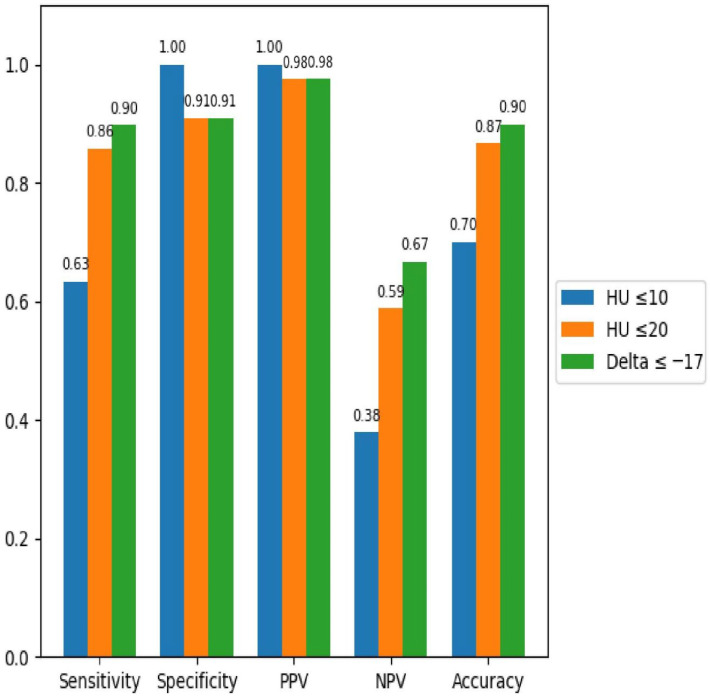
Bar plot of sensitivity, specificity, accuracy, PPV, and NPV for each diagnostic rule. The spectral attenuation difference (Δ40–140 keV ≤ −17 HU) showed the best overall diagnostic performance.

Diagnostic performance metrics are summarized in the bar plot (Figure 6), clearly showing that the spectral attenuation difference (Δ40–140 keV) provides the most balanced combination of sensitivity, specificity, and predictive values compared to the other evaluated rules.

## 4. Discussion

According to the 2023 European Society of Endocrinology (ESE) guidelines, unenhanced CT is considered the first imaging modality in the characterization of adrenal incidentalomas [19].

Our results support the role of conventional attenuation thresholds as reliable and well-established criteria for the characterization of adrenal lesions.

The 10 HU threshold is highly specific but less sensitive for the diagnosis of adrenal adenoma, while the 20 HU threshold improves sensitivity to the detriment of specificity [20].

Despite the very low prevalence of malignancy among homogeneous adrenal nodules <4 cm with attenuation between 10 and 20 HU, especially in patients without history of extra-adrenal malignancy, additional imaging or a 12-month follow-up are required.

The diagnostic accuracy of contrast-enhanced CT with a delayed washout (absolute and relative) is very low because up to one-third of pheochromocytomas and malignant tumors may show rapid washout similar to adenomas, while a notable fraction of benign adenomas do not meet the rapid washout criteria [20,21,22,23,24,25].

Finally, despite FDG-PET/CT representing the most reliable imaging method in the assessment of adrenal masses indeterminate at unenhanced CT, few malignant lesions are FDG-negative, especially renal cancer, and a subset of benign adenomas, especially if endocrine-active, are FDG-positive.

Dl-DECT has emerged as a useful tool for the characterization of incidental adrenal masses, because the ability to analyse lesion attenuation across a spectrum of energy levels provides crucial information on tissue composition [16].

Spectral analysis allows detection of fat within adenomas, similarly to MRI with chemical shift imaging, even when mean attenuation exceeds 10 HU, overcoming the main limitation of conventional CT.

Our data demonstrate that the attenuation difference between 40 keV and 140 keV VMIs offers superior diagnostic performance compared with conventional unenhanced attenuation thresholds.

The diagnostic rule based on a conventional unenhanced attenuation value ≤ 10 HU, traditionally used to identify lipid-rich adenomas, demonstrated a good discriminative ability, with an AUC of 0.82. Increasing the attenuation threshold to ≤ 20 HU resulted in an improvement in sensitivity for benign lesions, yielding an AUC of 0.88. However, this gain in sensitivity was accompanied by a higher false-positive rate.

The diagnostic rule based on the spectral attenuation difference between 40 keV and 140 keV VMIs (Δ40–140 keV ≤ −17 HU) exhibited the highest diagnostic accuracy, with an AUC of 0.90.

Although Δ40–140 keV yielded the highest area under the curve, formal comparison with continuous conventional attenuation using the DeLong test did not demonstrate a statistically significant difference. This finding is likely related, at least in part, to the limited sample size, particularly in the non-adenoma group. Nevertheless, bootstrap analysis confirmed the stability of the discriminative performance of the spectral parameter. Therefore, the proposed cutoff should be considered preliminary and deserving of validation in larger prospective cohorts.

Our results are consistent with prior literature. Nagayama et al., using ΔHU between 140 and 40 keV and 19 HU as the optimal threshold, demonstrated sensitivity and specificity of 76% and 97%, respectively, in a group of adrenal lesions with attenuation of 10–30 HU, potentially eliminating the need for additional diagnostic work-ups in this set of lesions [18].

Our study has several limitations that should be acknowledged. First, this is a retrospective, single-center study, which inherently limits the generalizability of the findings. Second, the overall sample size is relatively small, particularly with regard to patients with non-adenomatous lesions, potentially affecting the robustness of subgroup analyses.

Moreover, the study design involved the inclusion of only homogeneous lesions with adequate image quality, absence of significant artifacts, and a definitive final diagnosis in order to ensure reliable quantitative analysis.

In particular, a substantial number of clinically relevant malignant adrenal masses—such as metastases and adrenal carcinomas—were excluded due to their heterogeneous appearance, often related to necrosis, hemorrhage, or calcifications. The exclusion of inhomogeneous lesions may have led to an overestimation of the diagnostic performance of the evaluated approach, introducing a selection bias. However, according to current guidelines [2], only homogeneous adrenal masses are amenable to evaluation using unenhanced CT.

Larger, prospective, multicenter studies including a broader spectrum of adrenal lesions are warranted to validate these findings and better assess their applicability in routine clinical practice.

## 5. Conclusions

Unenhanced Spectral CT using the attenuation difference between 40 keV and 140 keV VMIs demonstrates higher diagnostic accuracy for differentiating adrenal adenomas from non-adenomatous lesions.

If further studies confirm our preliminary results, the integration of spectral parameters into diagnostic algorithms could reduce the number of indeterminate incidental adrenal lesions, avoiding the need for additional imaging in lesions with attenuation values between 10 HU and 20 HU.

## Figures and Tables

**Table 1:** CT acquisition parameters.

Parameters	Value
Tube voltage	120 kVp
Tube current	Automated modulation (Dose Right Index: 22)
Detector collimation	0.6 × 64 mm
Helical pitch	0.798
Rotation time	0.5 s

## Data Availability

The data presented in this study are available on request from the corresponding author due to ethical restrictions related to patient privacy.

## Abbreviations

The following abbreviations are used in this manuscript:

VMIs	virtual monoenergetic images
DECT	dual-energy CT
Dl-DECT	dual-layer DECT
